# Atraumatic Infected Septal Hematoma in a Pediatric Patient

**DOI:** 10.5811/cpcem.19476

**Published:** 2024-06-26

**Authors:** Osher Shefer, Jacqueline Le, Eshaan Daas, Eugene Hu

**Affiliations:** Desert Regional Medical Center, Department of Emergency Medicine, Palm Springs, California

**Keywords:** *nasal septal abscess*, *nasal septal hematoma*, *pediatric*, *atraumatic*, *ear-nose-throat (ENT)*

## Abstract

**Case Presentation:**

We present a case of a 10-year-old male who developed an atraumatic, nasal septal hematoma with abscess following several days of rhinorrhea and cough. His chief complaint to the emergency department was a two-day history of nasal swelling and discomfort, associated with difficulty breathing through his nose. The patient was well-appearing with swelling and tenderness along the external nasal ridge and nasal septal swelling that occluded both nares. Contrast-enhanced maxillofacial computed tomography revealed a rim-enhancing, fluid-filled collection to the anterior nasal septum. The patient underwent successful incision and drainage by otolaryngology.

**Discussion:**

Infected septal hematomas are rare but important to recognize as they can result in septal deformity and potentially life-threatening sequelae, such as intracranial infections. Most are secondary to nasal trauma in adult patients. This case highlights a unique presentation of atraumatic septal hematoma with abscess formation in an immunocompetent pediatric patient.

CPC-EM Capsule What do we already know about this clinical entity?

*Nasal septal abscess is a complication of traumatic septal hematoma with potential sequelae such as cartilage necrosis, cavernous sinus thrombosis, and meningitis.*

What is the major impact of the images?

*The images show atraumatic, infected septal hematoma in an immunocompetent pediatric patient, representing a rare clinical presentation in an uncommon demographic.*

How might this improve emergency medicine practice?

*Infected septal hematomas are rare and require timely diagnosis, which can be achieved with clinical suspicion guided by visual recognition of cardinal features.*

## CASE PRESENTATION

A 10-year-old male with no past medical or surgical history presented to our high-volume community emergency department with a two-day history of nasal swelling and discomfort associated with cough and rhinorrhea that began a few days prior. He had difficulty inhaling through his nose but denied any trauma, dental pain, fevers, or vomiting. No seasonal allergies or allergies to medications were reported by the patient’s family.

**Image 1.**
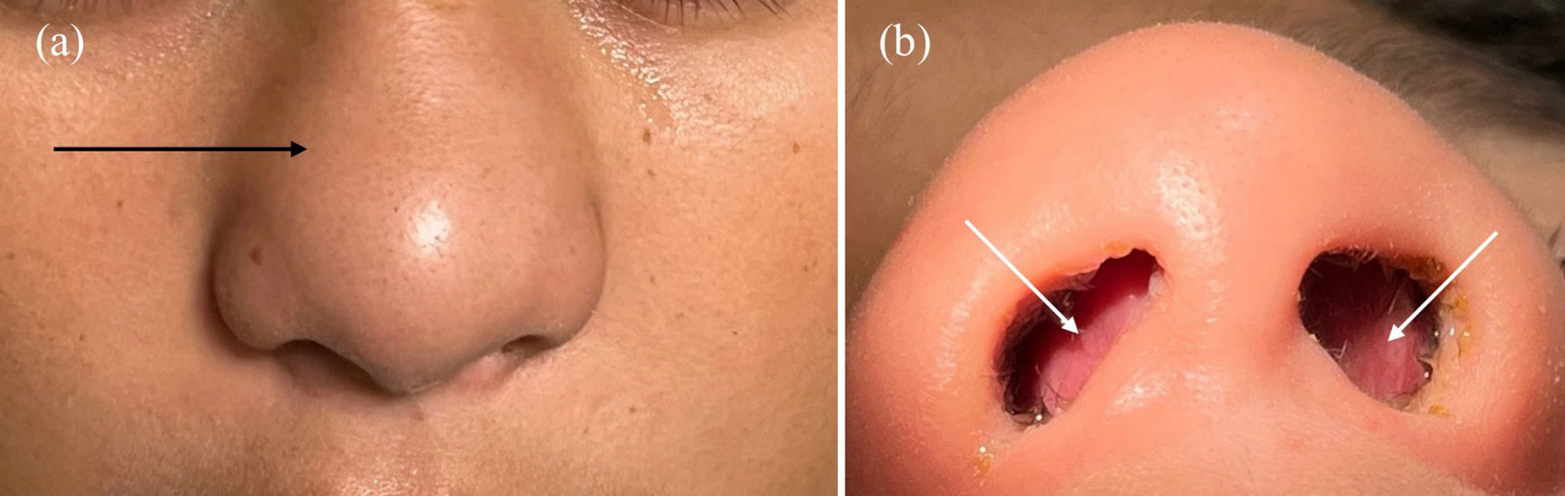
External nasal examination (a) of the patient demonstrated diffuse edema superior to both ala (black arrow). Internal nasal examination (b) revealed a markedly edematous nasal septum that extended and occluded both nares (white arrows). Clear rhinorrhea was noted.

The patient was well-appearing, with an elevated body mass index of 32 (reference range 18.5–24.9 for healthy weight). Vital signs were within normal limits, with temperature of 37.2° Celsius, heart rate of 95 beats per minute, respiratory rate of 18 breaths per minute, and oxygen saturation of 98% on room air. Physical examination revealed swelling and tenderness of the external aspect of the nose without evidence of skin lesions or trauma. The septum was edematous extending into and obstructing both nares, and clear nasal discharge was present (Image 1). He had no respiratory distress, and oropharynx was clear with normal dentition.

**Image 2.**
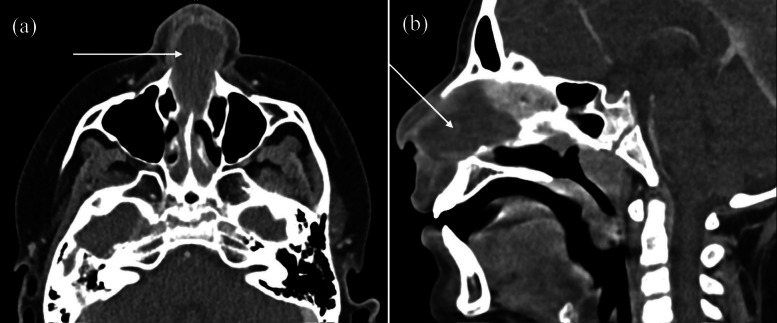
Axial (a) and sagittal (b) images of contrast-enhanced maxillofacial computed tomography demonstrating a large midline rim-enhancing, fluid-filled mass at the anterior nasal septum (arrows) with obstruction of both nasal cavities.

The otolaryngology (ENT) service was consulted, and they recommended maxillofacial computed tomography (CT) with contrast. Computed tomography imaging was significant for a 3.7 × 2.8 × 2.1 centimeter midline rim-enhancing, fluid collection at the anterior nasal septum with obstruction of the bilateral nasal cavity (Image 2). There was mild paranasal mucosal thickening without bony destruction. White blood cell count revealed a leukocytosis of 20.1 × 10^9^/liter (reference range 5.0–14.5 × 10^9^/liter) with left shift of 85.8% neutrophils (42–77%) and 10% lymphocytes (20–40%). Analgesia with acetaminophen and empiric intravenous antibiotics of vancomycin and piperacillin/tazobactam were initiated for suspected nasal septal abscess. The patient was transferred to a tertiary care facility with pediatric ENT services.

The following day, pediatric ENT performed an incision and drainage of the nasal septal collection with reported bloody output and scant purulence. Culture subsequently revealed light growth of *Staphylococcus aureus,* *Corynebacterium amycolatum,* and *S epidermidis.* The patient was discharged the day after surgery in stable condition without outpatient antibiotics.

## DISCUSSION

Nasal septal hematomas and abscesses are collections of blood or purulent material, respectively, within the nasal septum and are rare clinical occurrences. Nasal septal abscesses are almost exclusively described after trauma and are thought to arise secondary to septal hematoma formation.^1^ ^–^ ^4^ In one case series, all 20 pediatric patients who presented with septal hematoma and/or abscess had a reported history of nasal trauma.^1^ Less common causes of septal abscess include dental infection, rhinosinusitis, and postoperative sequelae.^2^ ^,^ ^3^ Typical pathogens include *S aureus, Streptococcus pneumoniae, Haemophilus influenzae,* and anaerobic bacteria.^2^ Atraumatic nasal septal abscesses have been more commonly reported in immunocompromised adults, and a spontaneous nasal septal hematoma with abscess has been reported in an adult.^5^ Our case is novel in that we present an atraumatic, infected septal hematoma in an immunocompetent pediatric patient.

A clinical presentation of pain and swelling with examination findings seen in Images 1 and 2 should prompt consideration of septal hematoma or abscess. In our case ENT recommended advanced imaging and transfer to a site with pediatric ENT availability. Timely recognition and diagnosis is pivotal to optimal patient outcomes and minimizing potential complications including nasal deformity, septal perforation, and intracranial infection.^2^
